# Can lymphovascular invasion be predicted by contrast-enhanced CT imaging features in patients with esophageal squamous cell carcinoma? A preliminary retrospective study

**DOI:** 10.1186/s12880-022-00804-7

**Published:** 2022-05-17

**Authors:** Yang Li, Haiyan Su, Li Yang, Meng Yue, Mingbo Wang, Xiaolong Gu, Lijuan Dai, Xiangming Wang, Xiaohua Su, Andu Zhang, Jialiang Ren, Gaofeng Shi

**Affiliations:** 1 Department of Computed Tomography and Magnetic Resonance, Fourth Hospital of Hebei Medical University, Shijiazhuang, 050011 China; 2 Department of Pathology, Fourth Hospital of Hebei Medical University, Shijiazhuang, 050011 China; 3 Department of Thoracic Surgery, Fourth Hospital of Hebei Medical University, Shijiazhuang, 050011 China; 4 Department of Oncology, Hebei General Hospital, Shijiazhuang, 050051 China; 5 Department of Radiotherapy, Fourth Hospital of Hebei Medical University, Shijiazhuang, 050011 China; 6 GE Healthcare China, Beijing, 100176 China

**Keywords:** Contrast-enhanced computed tomography, Lymphovascular invasion, CT attenuation value, Predictor

## Abstract

**Background:**

To investigate the value of contrast-enhanced CT (CECT)-derived imaging features in predicting lymphovascular invasion (LVI) status in esophageal squamous cell carcinoma (ESCC) patients.

**Methods:**

One hundred and ninety-seven patients with postoperative pathologically confirmed esophageal squamous cell carcinoma treated in our hospital between January 2017 and January 2019 were enrolled in our study, including fifty-nine patients with LVI and one hundred and thirty-eight patients without LVI. The CECT-derived imaging features of all patients were analyzed. The CECT-derived imaging features were divided into quantitative features and qualitative features. The quantitative features consisted of the CT attenuation value of the tumor (CTV_Tumor_), the CT attenuation value of the normal esophageal wall (CTV_Normal_), the CT attenuation value ratio of the tumor-to-normal esophageal wall (TNR), the CT attenuation value difference between the tumor and normal esophageal wall (ΔTN), the maximum thickness of the tumor measured by CECT (Thickness), the maximum length of the tumor measured by CECT (Length), and the gross tumor volume measured by CECT (GTV). The qualitative features consisted of an enhancement pattern, tumor margin, enlarged blood supply or drainage vessels to the tumor (EVFDT), and tumor necrosis. For the clinicopathological characteristics and CECT-derived imaging feature analysis, the chi-squared test was used for categorical variables, the Mann–Whitney U test was used for continuous variables with a nonnormal distribution, and the independent sample t-test was used for the continuous variables with a normal distribution. The trend test was used for ordinal variables. The association between LVI status and CECT-derived imaging features was analyzed by univariable logistic analysis, followed by multivariable logistic regression and receiver operating characteristic (ROC) curve analysis.

**Results:**

The CTV_Tumor_, TNR, ΔTN, Thickness, Length, and GTV in the group with LVI were higher than those in the group without LVI (*P* < 0.05). A higher proportion of patients with heterogeneous enhancement pattern, irregular tumor margin, EVFDT, and tumor necrosis were present in the group with LVI (*P* < 0.05). As revealed by the univariable logistic analysis, the CECT-derived imaging features, including CTV_Tumor_, TNR, ΔTN and enhancement pattern, Thickness, Length, GTV, tumor margin, EVFDT, and tumor necrosis were associated with LVI status (*P* < 0.05). Only the TNR (OR 8.655; 95% CI 2.125–37.776), Thickness (OR 6.531; 95% CI 2.410–20.608), and tumor margin (OR 4.384; 95% CI 2.004–9.717) were independent risk factors for LVI in the multivariable logistic regression analysis. The ROC curve analysis incorporating the above three CECT-derived imaging features showed that the area under the curve obtained by the multivariable logistic regression model was 0.820 (95% CI 0.754–0.885).

**Conclusion:**

The CECT-derived imaging features, including TNR, Thickness, tumor margin, and their combination, can be used as predictors of LVI status for patients with ESCC.

**Supplementary Information:**

The online version contains supplementary material available at 10.1186/s12880-022-00804-7.

## Introduction

Globally, esophageal carcinoma ranks seventh in incidence and sixth in mortality overall [1].Esophageal carcinoma has a poor prognosis and the fourth highest mortality rate in China, with more than 200,000 deaths per year [2].The long-term postoperative outcomes are far from satisfactory, with previous studies reporting recurrence rates between 42 and 52% after radical resection [3, 4]. Despite multimodality therapy, the prognosis for patients with esophageal carcinoma remains poor in Eastern Asia [5]. Therefore, accurate preoperative risk assessment and stratification are necessary for optimal treatment planning.

Lymphovascular invasion (LVI) is a histopathological feature associated with biologically aggressive disease, and LVI is easily and reliably assessed using routine light microscopy [6]. Indeed, LVI is a necessary and important step in lymph node metastasis and for the systemic dissemination of cancer cells, and it is thought to increase the risk of micrometastasis in localized cancers [7]. A growing number of retrospective studies that have evaluated the relationship between LVI and prognosis have demonstrated that esophageal squamous cell carcinoma (ESCC) patients with LVI have a poor prognosis [6, 8–10].

Several studies have found that tumors with LVI are larger in size, length, and depth of infiltration than those without LVI, thus suggesting an expanded scope of resection and lymph node dissection or a combination of adjuvant treatments [11, 12]. For patients with ESCC undergoing endoscopic resection, additional surgery or prophylactic chemoradiotherapy is indicated if LVI is detected, even if there are no clinical signs of metastasis [13]. A valid and sound management decision based on LVI can lead to good long-term results [14]. Consequently, effective systemic therapy and intensive monitoring are necessary for ESCC patients with LVI [15].

Previously, Ma et al. [16] found that multiphase dynamic CT provides a noninvasive method for predicting advanced gastric cancer (AGC) in LVI with quantitative enhancement measurements. Yin et al. [17] showed that the contrast enhancement ratio (CER) of triple-phase multislice CT scans in gastric cancer is closely correlated with intratumoral microvascular and lymphatic invasion, and the CER could be used as a marker for histological differentiation. To the best of our knowledge, there have been no studies using contrast-enhanced CT (CECT)-derived imaging features, such as CT attenuation value-derived parameters and morphological features, to predict LVI status in patients with ESCC.

Therefore, our study aimed to investigate the value of CECT-derived imaging features in predicting LVI status in ESCC patients and provide a potential preoperative risk stratification for optimal treatment planning.

## Methods

### Patients

**Fig. 1:**
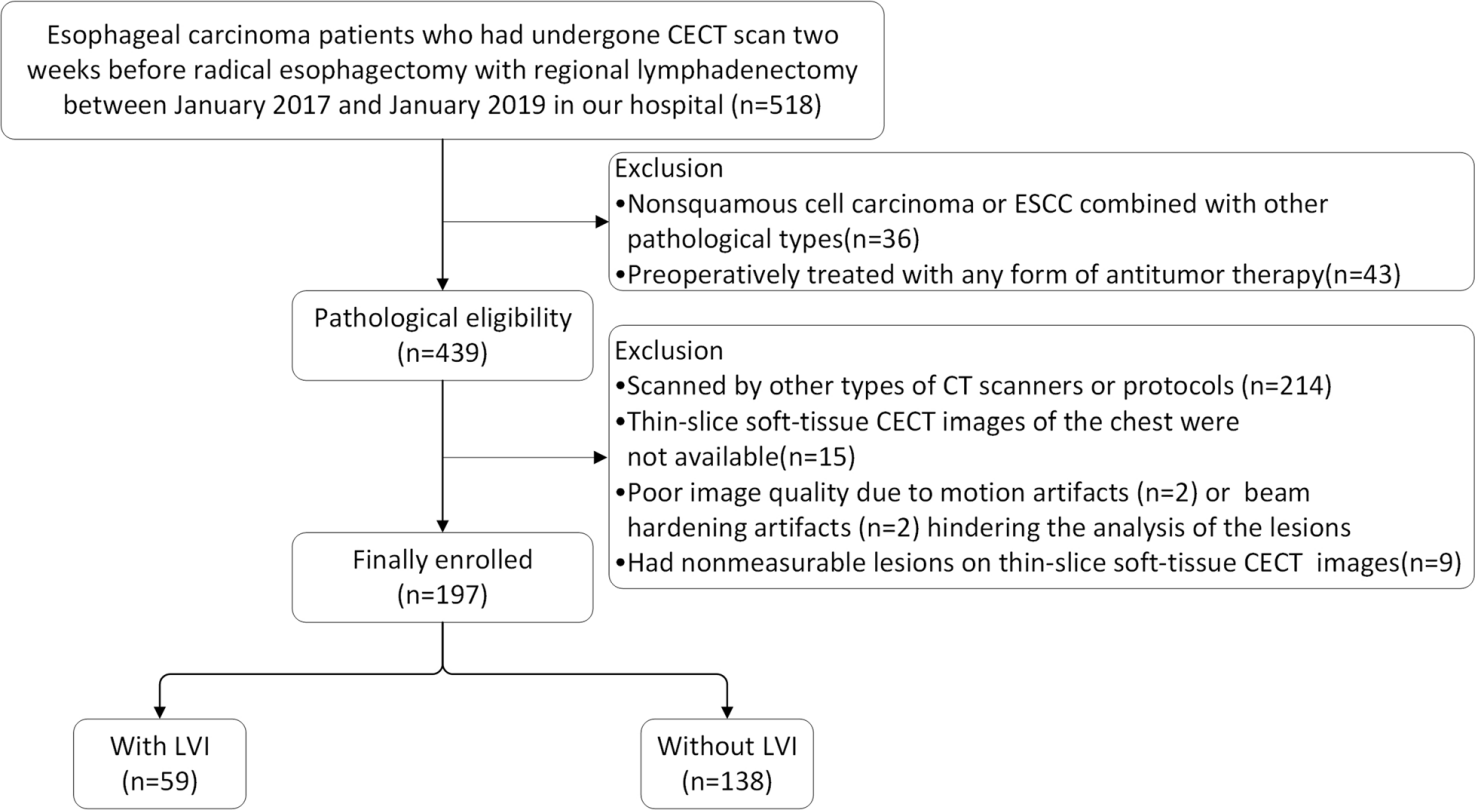
Flow chart showing the patient selection process and exclusion criteria

This present retrospective study was approved by the ethics committee of our hospital, and the requirement for written informed consent was waived. A total of 518 consecutive patients with esophageal carcinoma who underwent CECT scans two weeks before radical esophagectomy with regional lymphadenectomy between January 2017 and January 2019 in our hospital were analyzed. The inclusion criteria for the enrolled patients were as follows: ❶ had pathologically confirmed esophageal carcinoma patients who had undergone radical esophagectomy and regional lymphadenectomy; ❷ underwent a CECT scan of the chest or the chest plus upper abdomen within 2 weeks before surgery; The exclusion criteria were as follows: ❶ were nonsquamous cell carcinoma or ESCC combined with other pathological types (n = 36); ❷ preoperatively treated with any form of antitumor therapy(n = 43); ❸ scanned by other types of CT scanners or protocols (n = 214). ❹ thin-slice soft-tissue CECT images of the chest were not available(n = 15); ❺ poor image quality due to motion artifacts (n = 2) or beam hardening artifacts (n = 2) hindering the analysis of the lesions; and ❻ had nonmeasurable lesions on thin-slice soft-tissue CECT images(n = 9). Finally, 197 patients (age range: 42–77 years; mean age: 62.53 years) with postoperative pathologically confirmed ESCC were included in this study (Fig. 1).

### CT protocol

All enrolled patients were scanned with a second-generation dual-source CT scanner (Somatom Definition Flash; Siemens, Germany). The scanning parameters were as follows: a tube voltage of 120 kVp, an automatic mA, a slice thickness of 5.0 mm, increments of 5.0 mm, a rotation time of 0.5 s, a pitch of 1.2, a matrix of 512 × 512, a soft-tissue reconstruction kernel of B30f, and a reconstruction slice thickness of 1.0 mm. No anticholinergic drugs were used in the present study. All patients were asked to fast for approximately 4–6 h and had breathing training before the CT examination. To clean and dilate the esophagus, all patients were asked to drink 500–1000 ml of pure water 1–5 min before the examination. The arterial phase CT scans were performed with a 30 s delay after intravenous injection of contrast medium (3.0–4.0 ml/s, 1.5 ml/kg, 300 mg I/ml, Iohexol) via a syringe pump, followed by a 20 ml saline flush.

### CECT-derived imaging feature analysis

The thin-slice CECT images were exported from the picture archiving and communication system (PACS) in the DICOM format. All the thin-slice soft-tissue CECT images were analyzed independently by two radiologists (radiologist 1 and radiologist 2) with 10 years of experience in the CT diagnosis of esophageal carcinoma. The two radiologists were only aware that all of the cases were ESCC patients and were blinded to all of the other clinical and pathological information. The criteria for a lesion included local or circumferential thickening of the esophageal wall greater than 5 mm with abnormal enhancement, a gas-free esophageal wall that was greater than 10 mm in diameter, or irregular local lumen stenosis [18].Before starting the analysis, the two observers discussed and defined the measurement and characteristics of CECT-derived imaging features. The CECT-derived imaging features were divided into quantitative features and qualitative features. The quantitative consisted of the CT attenuation value of the tumor (CTV_Tumor_), the CT attenuation value of the normal esophageal wall (CTV_Normal_), the CT attenuation value ratio of the tumor-to-normal esophageal wall (TNR), the CT attenuation value difference between the tumor and normal esophageal wall (ΔTN), the maximum thickness of the tumor measured by CECT (Thickness), the maximum length of the tumor measured by CECT (Length), and the gross tumor volume measured by CECT (GTV). The qualitative features consisted of enhancement pattern, tumor margin, enlarged blood supply or drainage vessels to the tumor (EVFDT), and tumor necrosis.

**Fig. 2:**
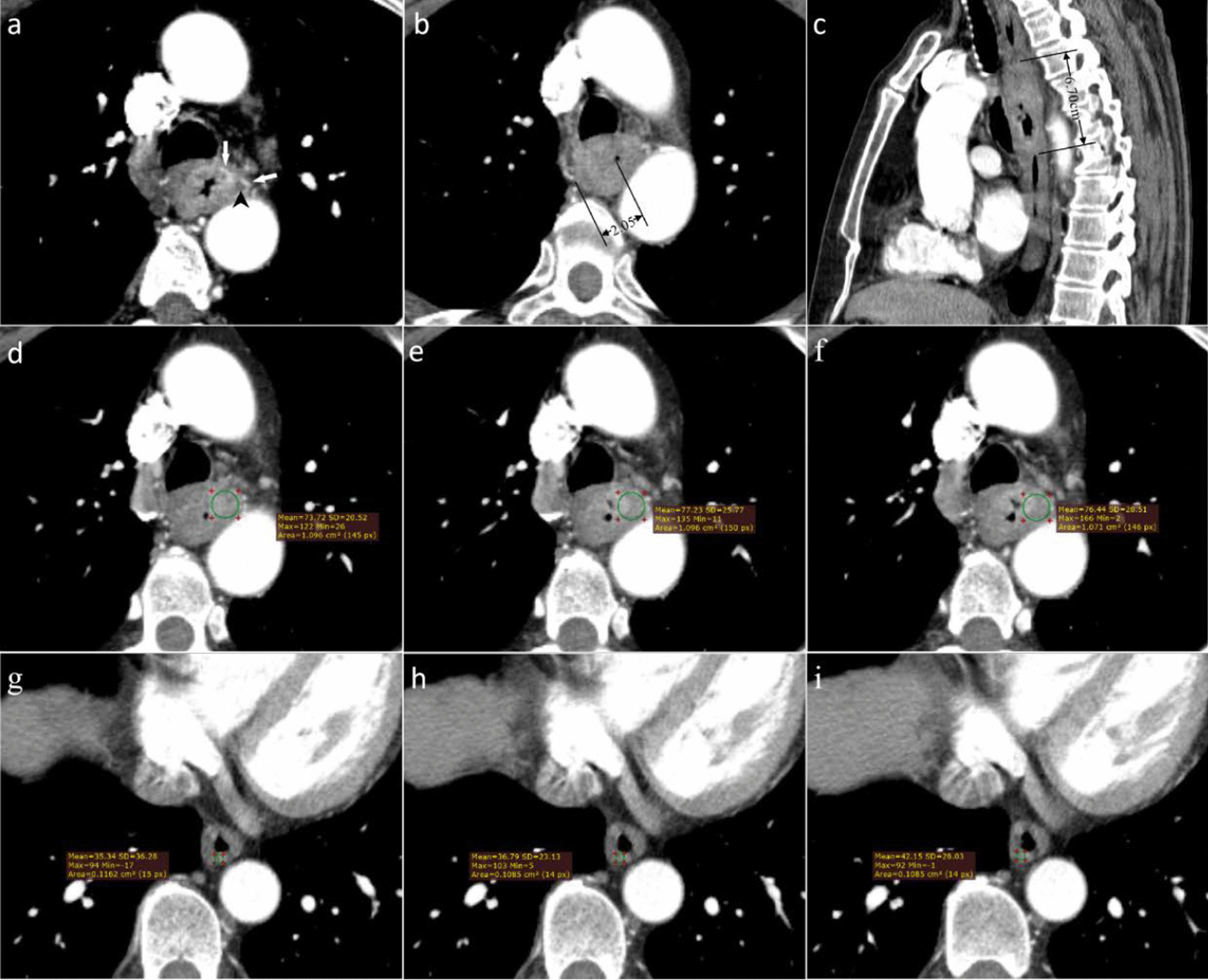
CECT-derived imaging feature analysis for a 60-year-old male ESCC patient with LVI. The tumor was located in the upper esophagus and had a postoperative pathological stage of pT3N2M0 (G3). **a** Axial CECT image shows a tumor with heterogeneous enhancement and irregular tumor margin. EVFDT (white arrows) is visible in the interior and at the tumor margin. Tumor necrosis (black arrowhead) appears as hypodense foci within the tumor. **b** The maximum thickness of the tumor measured on axial CECT image was 2.05 cm and an adjacent eccentrically narrowed lumen (black arrow) can be seen. **c** The maximum length of the tumor was 6.70 cm, as measured on the sagittal image. **d**–**f** The CTV_Tumor_ was measured by placing ROIs (green circle) at the 3 consecutive levels with the most pronounced tumor enhancement. The CTV_Tumor_ was 75.80 HU = (73.72HU + 77.23HU + 76.44HU)/3. **g**–**i** The mean CT attenuation value of the normal esophageal wall was measured by placing ROIs (green circle) at 3 consecutive levels with the homogenous enhancement. The CTV_Normal_ was 38.02 HU = (35.14HU + 36.79HU + 42.15HU)/3. The ΔTN was 37.78HU = 75.80 HU-38.02 HU. The TNR was 1.99 = 75.80HU/38.02 HU

**Fig. 3:**
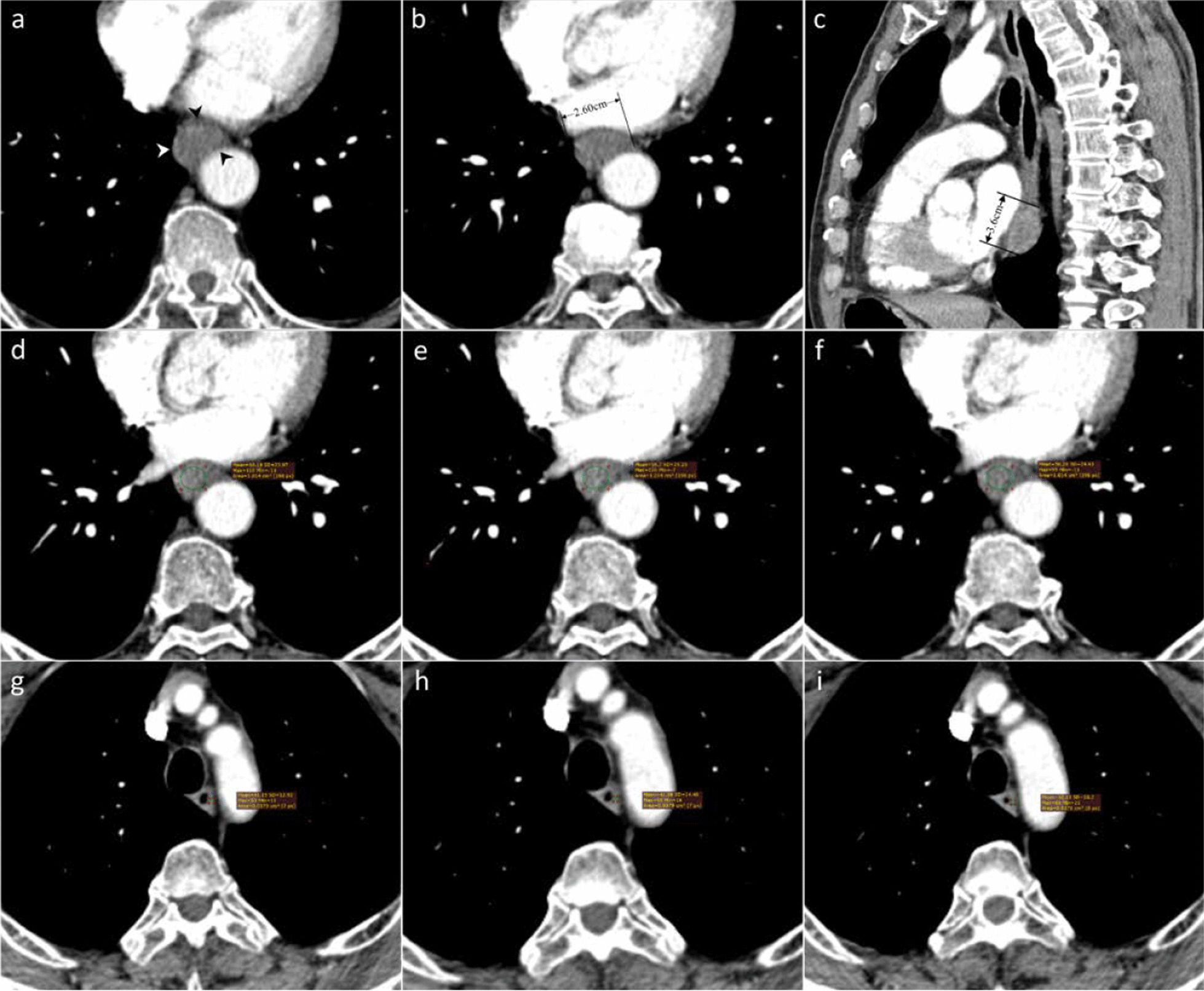
CECT-derived imaging feature analysis for a 70-year-old male ESCC patient without LVI. The tumor was located in the lower esophagus and had a postoperative pathological stage of pT3N0M0 (G2). **a** Axial CECT image shows a tumor with homogeneous enhancement and regular margin (white and black arrows) without EVFDT or tumor necrosis. **b** The tumor narrowed the lumen, and the Thickness is 1.3 cm (2.6 cm/2). **c** The maximum length of the tumor was 3.60 cm, as measured on the sagittal image. **d**–**f** The CTV_Tumor_ was 55.72HU = (60.19HU + 56.70HU + 50.28HU)/3. **g**–**i** The CTV_Normal_ was 41.62 HU = (41.15HU + 41.58HU + 42.13HU)/3. The ΔTN was 14.1HU = 55.72 HU-41.62 HU. The TNR was 1.39 = 55.72HU/41.62HU

First, the thin-slice CECT images of all patients were uploaded into the RadiAnt DICOM viewer (open-source software, https://www.radiantviewer.com/). The multi-planar reconstruction (MPR) mode was used to maximally display the entire tumor in the sagittal position. On axial images, the circular regions of interest (ROIs) of approximately 8–25 mm in diameter were placed in the area of most pronounced enhancement at the 3 consecutive levels (Figs. 2d–f, 3d–f). The ROIs were maximized to cover as much of the most distinctly enhanced area as possible without exceeding the tumor boundary. As much as possible, the ROIs were drawn to avoid ulcers and necrosis within the tumor, gas in the lumen, blood vessels, and the fat tissue surrounding the tumor. The ROIs of the normal esophageal wall were placed more than 5 cm away from the tumor edge (Figs. 2g–i, 3g–i). The ROIs included as much of the normal esophagus as possible while avoiding structures such as gas in the lumen and surrounding fat and blood vessels. Measurements were performed at three successive levels. Meanwhile, the CTV_Tumor_ and CTV_Normal_ in Hounsfield units (HU) of the ROIs were recorded. The TNR and ΔTN were calculated according to the following formulae:

$$\begin{aligned} & {\text{TNR}} = {\text{CTV}}_{{{\text{Tumor}}}} {\text{/CTV}}_{{{\text{Normal}}}} \\ & \Delta {\text{TN}} = {\text{CTV}}_{{{\text{Tumor}}}} {-}{\text{CTV}}_{{{\text{Normal}}}} . \\ \end{aligned}$$

The enhancement pattern was categorized as homogeneous or heterogeneous (Figs. 2a, 3a). The Thickness was obtained on axial images (Figs. 2b, 3b), while the Length was obtained on sagittal images (Figs. 2c, 3c). The presence of peripheral or central EVFDT was assessed as previously described [19]. The presence of low intratumoral attenuation was considered to be necrosis when the CT attenuation value was < 20 HU [20].

**Fig. 4:**
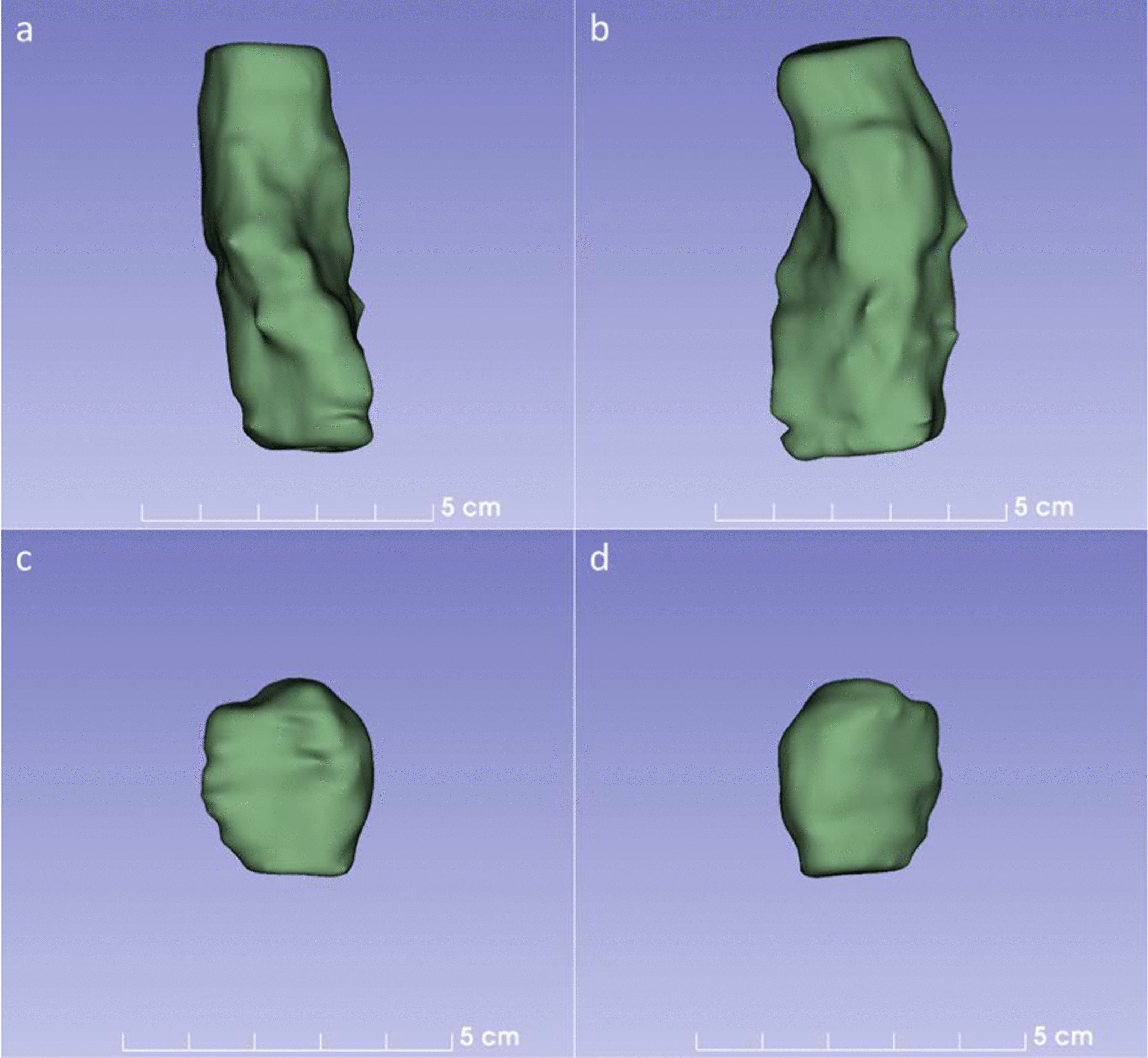
The GTV of the tumor was obtained by using 3D Slicer software. **a**, **b** The GTV was derived from a patient with LVI in Fig. 2. The GTV was 20.60 cm^3^ with an irregular tumor margin. **c**, **d** The GTV was derived from a patient without LVI in Fig. 3. The GTV was 5.06 cm^3^ with a regular tumor margin

Second, the thin-slice CECT images were then transferred into 3D Slicer software (Version 4.10.2, open-source software, http://www.slicer.org/), and the tumors were manually outlined layer by layer to obtain the GTV (Fig. 4). The tumor margin was classified as regular or irregular. The evaluation was based on axial images, referring to the MPR images and stereoscopic three-dimensional (3D) images were from all angles. If the tumor margin was well defined, the surrounding fat space was present on axial images, and the surface was relatively smooth on 3D images, it was classified as a regular tumor margin. If the tumor margin was not well defined, the surrounding fat space was partially or entirely disappeared on axial images, and multiple bumps were visible on axial images on the surface on 3D images, then it was classified as an irregular tumor margin.

Intraclass correlation coefficient (ICC) analysis and Cohen's kappa analysis were used to assess interobserver agreement between radiologist 1 and radiologist 2. Two weeks later, all of the measurements and evaluations were repeated by radiologist 1 in the same manner but in a random order to verify the intraobserver agreement. If the agreements were good, the mean values of the two observers' measurements were taken for subsequent analysis of quantitative data. For categorical data, the final results were determined by mutual agreement between the two observers and then used for the subsequent analysis.

### Pathological evaluation

All enrolled patients underwent surgery within 2 weeks after the CECT examination. All surgically resected tumor specimens were processed according to the standard pathological procedures and were examined by two experienced pathologists (with 11 and 9 years of experience in esophageal pathology). LVI is defined as the presence of tumor emboli within the arterial, venous, or lymphatic vessels.

### Statistical analysis

In this study, the patients were divided into two groups: patients with LVI and patients without LVI. Continuous variables that conformed to a normal distribution are expressed as M ± SD, and those that did not conform to a normal distribution are expressed as median (interquartile range). For the clinicopathological characteristics and CECT-derived imaging feature analysis, the chi-squared test was used for categorical variables, the Mann–Whitney U test was used for continuous variables with a nonnormal distribution, and the independent sample t-test was used for the continuous variables with a normal distribution. The trend test was used for ordinal variables. The reported significance levels were all two-sided and were set at 0.05.

The ICC analysis was used to assess the intraobserver and interobserver agreements for quantitative analysis. The ICC value was interpreted as follows: poor agreement for ICC ≤ 0.50; moderate agreement for 0.50 < ICC ≤ 0.75; good agreement for 0.75 < ICC ≤ 0.90; excellent agreement for ICC > 0.90 [21]. Cohen's kappa analysis was used to assess the intraobserver and interobserver agreements for qualitative analysis. The kappa value was interpreted as follows: a kappa value of 0.20–0.40 indicating fair agreement; 0.41–0.60, moderate agreement; 0.61–0.80, good agreement; and greater than 0.80, excellent agreement [22].

The CECT-derived imaging features with statistically significant differences between the two groups were incorporated into the univariable logistic regression analysis. In the univariable logistic regression analysis, the variables with *P* < 0.05 were considered to be associated with LVI status and were incorporated into the multivariable analysis. The independent predictors for LVI were identified, and their combination was built by stepwise multivariable logistic regression analysis. In addition, receiver operating characteristic (ROC) curves were plotted, and the area under the curve (AUC), accuracy, sensitivity, specificity, positive predictive value (PPV), and negative predictive value (NPV) were calculated. A nomogram was formulated based on the multivariable logistic regression analysis. All statistical analysis was performed using MedCalc Statistical Software (Version 20.022; https://www.medcalc.org/) and R software (Version: 3.6.3; http://www.r-project.org/).

## Results

### Clinicopathological characteristics of the patients

The clinicopathological characteristics of the 197 enrolled patients are presented in Table 1. There were 59 patients with LVI (29.9%) and 138 patients without LVI (70.01%).

**Table 1:** Clinicopathological characteristics of all enrolled ESCC patients

Variables	With LVI(n = 59)	Without LVI (n = 138)	Total (n = 197)	*P*
Sex, n (%)				0.087^#^
Male	45 (76.3%)	88 (63.8%)	133 (67.5%)	
Female	14 (23.7%)	50 (36.2%)	64 (32.5%)	
Age, n (%)				0.495^#^
< 60	24 (40.7%)	49 (35.5%)	73 (37.1%)	
≥ 60 years	35 (59.3%)	89 (64.5%)	124 (62.9%)	
Location, n (%)				0.963^#^
Upper	4 (6.78%)	10 (7.25%)	14 (7.11%)	
Middle	44 (74.6%)	99 (71.7%)	143 (72.6%)	
Lower	11 (18.6%)	29 (21.0%)	40 (20.3%)	
pThickness (cm)	1.30(1.00–1.75)	1.00 (0.80–1.40)	1.00 (1.00–1.50)	< 0.001^*^
pLength (cm)	4.00 (3.00–5.00)	3.50 (3.00–4.38)	3.50 (3.00–5.00)	0.001^*^
PNI, n (%)				0.020^#^
Negative	31 (52.5%)	98 (71.0%)	129 (65.5%)	
Positive	28 (47.5%)	40 (29.0%)	68 (34.5%)	
Differentiation grade, n (%)				0.177^&^
I	0 (0.00%)	2 (1.45%)	2 (1.02%)	
II	34 (57.6%)	96 (69.6%)	130 (66.0%)	
III	25 (42.4%)	40 (29.0%)	65 (33.0%)	
pT stage, n (%)				< 0.001^&^
T1	0	12 (8.70%)	12 (6.09%)	
T2	3 (5.08%)	33 (23.9%)	36 (18.3%)	
T3	56 (94.9%)	92 (66.7%)	148 (75.1%)	
T4	0 (0.00%)	1 (0.72%)	1 (0.51%)	
pN stage, n (%)				< 0.001^&^
N0	10 (16.9%)	89 (64.5%)	99 (50.3%)	
N1	21 (35.6%)	32 (23.2%)	53 (26.9%)	
N2	15 (25.4%)	14 (10.1%)	29 (14.7%)	
N3	13 (22.0%)	3 (2.17%)	16 (8.12%)	
pAJCC stage, n (%)				< 0.001^&^
I	0	8 (5.80%)	8 (4.06%)	
II	10 (16.9%)	83 (60.1%)	93 (47.2%)	
III	33 (55.9%)	43 (31.2%)	76 (38.6%)	
IV	16 (27.1%)	4 (2.90%)	20 (10.2%)	

^#^Chi-square test

*Mann–Whitney U test

^&^Trend test

With regard to sex, age, and tumor location, there were no significant differences between the two groups.

In the group with LVI, the maximum tumor thickness and length in gross pathology were greater than those in the group without LVI (*P* < 0.05). Compared to tumors in the group without LVI, tumors in the group with LVI had a higher pT stage, pN stage, and pAJCC stage (all *P* < 0.05). The incidence of perineural invasion (PNI) in tumors with LVI was 71%, which was higher than the incidence of PNI in tumors without LVI (*P* = 0.020). The detailed results are given in Table 1.

### Intraobserver and interobserver agreements

For the measurement and evaluation of the CECT-derived imaging features, no significant differences were observed between radiologist 1 and radiologist 1 (*P* = 0.057–0.989) or between radiologist 1 and radiologist 2 (*P* = 0.100–0.947). The ICC and kappa values for the CT-derived imaging features ranged from 0.792 to 0.986. This result indicated that the intraobserver and interobserver agreements were good or excellent. The detailed results are given in Additional file 1: Tables S1 and S2.

### Comparison of the CECT-derived imaging features between the two groups

The results of the univariable analysis of the preoperative CECT-derived imaging features are presented in Table 2. The CTV_Tumor_ in the patients with LVI was significantly higher than that in the patients without LVI (*P* < 0.001). With regard to CTV_Normal_, there was no significant difference between the two groups (*P* = 0.413). The TNR and ΔTN in the group with LVI were higher than those without LVI (*P* = 0.001; *P* < 0.001). The CECT-derived imaging features, such as Thickness, Length, and GTV were greater in the group with LVI (all *P* < 0.001). A higher proportion of patients with irregular tumor margin (57.6%, 34/59), EVFDT (61.0%, 36/59), and tumor necrosis (57.6%, 17/59) were present in the group with LVI (all *P* < 0.001).

**Table 2:** The CECT-derived imaging feature analysis of the enrolled ESCC patients

Variables	With LVI(n = 59)	Without LVI (n = 138)	Total (n = 197)	*P*
CTV_Tumor_ (HU)	72.0 (66.5–79.0)	65.0 (58.2–72.8)	68.0 (61.0–75.0)	< 0.001^#^
CTV_Normal_ (HU)	40.6 ± 4.9	40.0 ± 5.1	40.2 ± 5.1	0.413^&^
TNR	1.79 (1.64–1.90)	1.64 (1.47–1.85)	1.68 (1.51–1.88)	0.001^#^
**Δ**TN (HU)	32.0(27.0–37.0)	25.0 (18.0–33.8)	27.0 (21.0–34.0)	< 0.001^#^
Enhancement pattern				0.001*
Homogenous	42 (71.2%)	125 (90.6%)	167 (84.8%)	
Heterogeneous	17 (28.8%)	13 (9.42%)	30 (15.2%)	
Thickness (cm)	1.65 (1.42–2.05)	1.40 (1.20–1.55)	1.47 (1.25–1.67)	< 0.001^#^
Length (cm)	5.36 (4.48–6.03)	4.12 (3.42–4.94)	4.43 (3.71–5.46)	< 0.001^#^
GTV (cm^3^)	14.8 (11.7–26.2)	8.34 (4.23–14.3)	10.8(5.70–16.7)	< 0.001^#^
Tumor margin, n (%)				< 0.001*
Regular	25 (42.4%)	117 (84.8%)	142 (72.1%)	
Irregular	34 (57.6%)	21 (15.2%)	55 (27.9%)	
EVFDT, n (%)				< 0.001*
Absent	23 (39.0%)	98 (71.0%)	121 (61.4%)	
Present	36 (61.0%)	40 (29.0%)	76 (38.6%)	
Necrosis, n (%)				0.007*
Absent	42 (71.2%)	121 (87.7%)	163 (82.7%)	
Present	17 (28.8%)	17 (12.3%)	34 (17.3%)	

^#^Mann–Whitney U test

^&^Independent sample t test

*Chi-square test

### Univariable and multivariable logistic regression analysis

The results of the univariable and multivariable regression logistic analysis for CECT-derived imaging features are presented in Table 3. In the univariable analysis, CTV_Tumor_, TNR, **Δ**TN, enhancement pattern, Thickness, Length, GTV, tumor margin, EVFDT, and tumor necrosis were associated with LVI status (all *P* < 0.05). However, in multivariable analysis, Thickness, ΔTN, and tumor margin were independent predictors of LVI (all *P* < 0.05).

**Table 3:** Univariable and multivariable logistic regression analysis of the CECT-derived imaging features

Variables	Univariable		Multivariable	
	OR (95% CI)	*P*	OR (95% CI)	*P*
CTV_Tumor_	1.071 (1.036–1.108)	< 0.001	–	–
TNR	8.300 (2.404–28.659)	0.001	8.655 (2.125–37.776)	0.003
**Δ**TN	1.080 (1.041–1.121)	< 0.001	–	–
Enhancement pattern			–	–
Homogenous	Reference		–	–
Heterogeneous	3.850 (1.724–8.801)	0.001	–	–
Thickness	13.495 (4.817–37.808)	< 0.001	6.531 (2.410–20.608)	0.001
Length	1.959 (1.499–2.560)	< 0.001	–	–
GTV	1.089 (1.049–1.129)	< 0.001	–	–
Tumor margin			–	–
Regular	Reference		Reference	
Irregular	7.450 (3.756–15.229)	< 0.001	4.384 (2.004–9.717)	0.001
EVFDT			–	–
Absent	Reference		–	–
Present	3.796 (2.013–7.305)	< 0.001	–	–
Necrosis			–	–
Absent	Reference		–	–
Present	2.862 (1.329–6.186)	0.007	–	–

### Diagnostic performance of preoperative CECT-derived imaging features and their combination

**Fig. 5:**
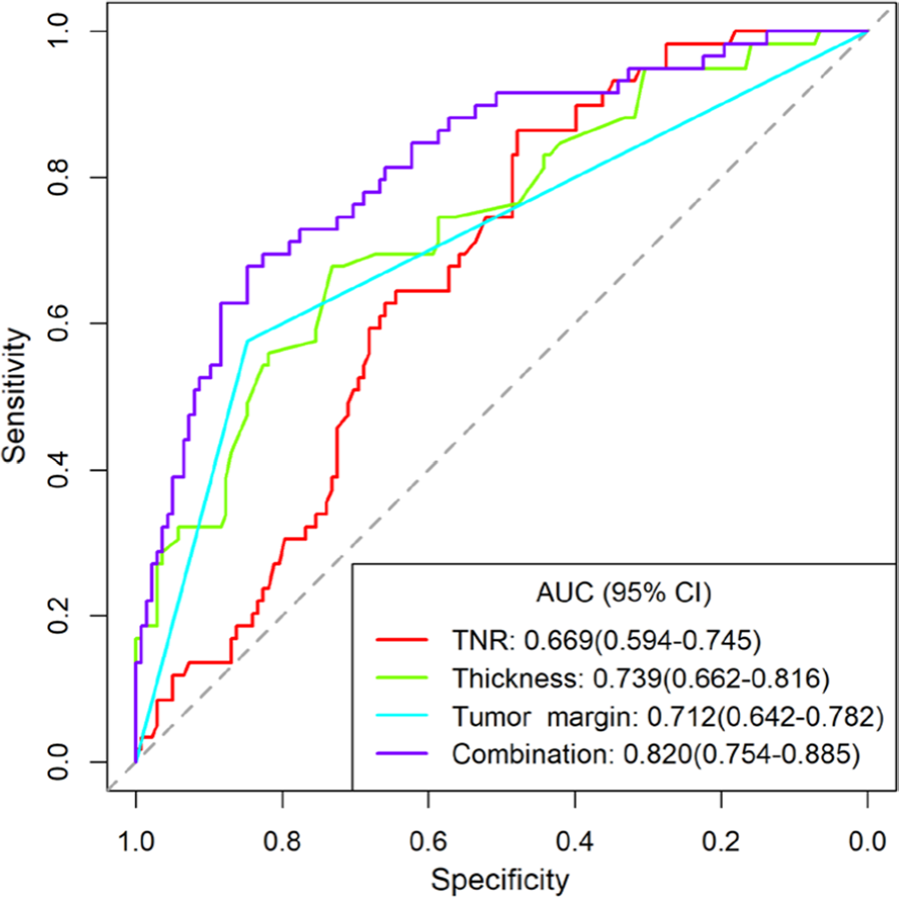
Receiver operator characteristic (ROC) analysis for predicting LVI status. The AUC values of the TNR, Thickness, tumor margin, and their combination were 0.669, 0.739, 0.712, and 0.820, respectively

**Fig. 6:**
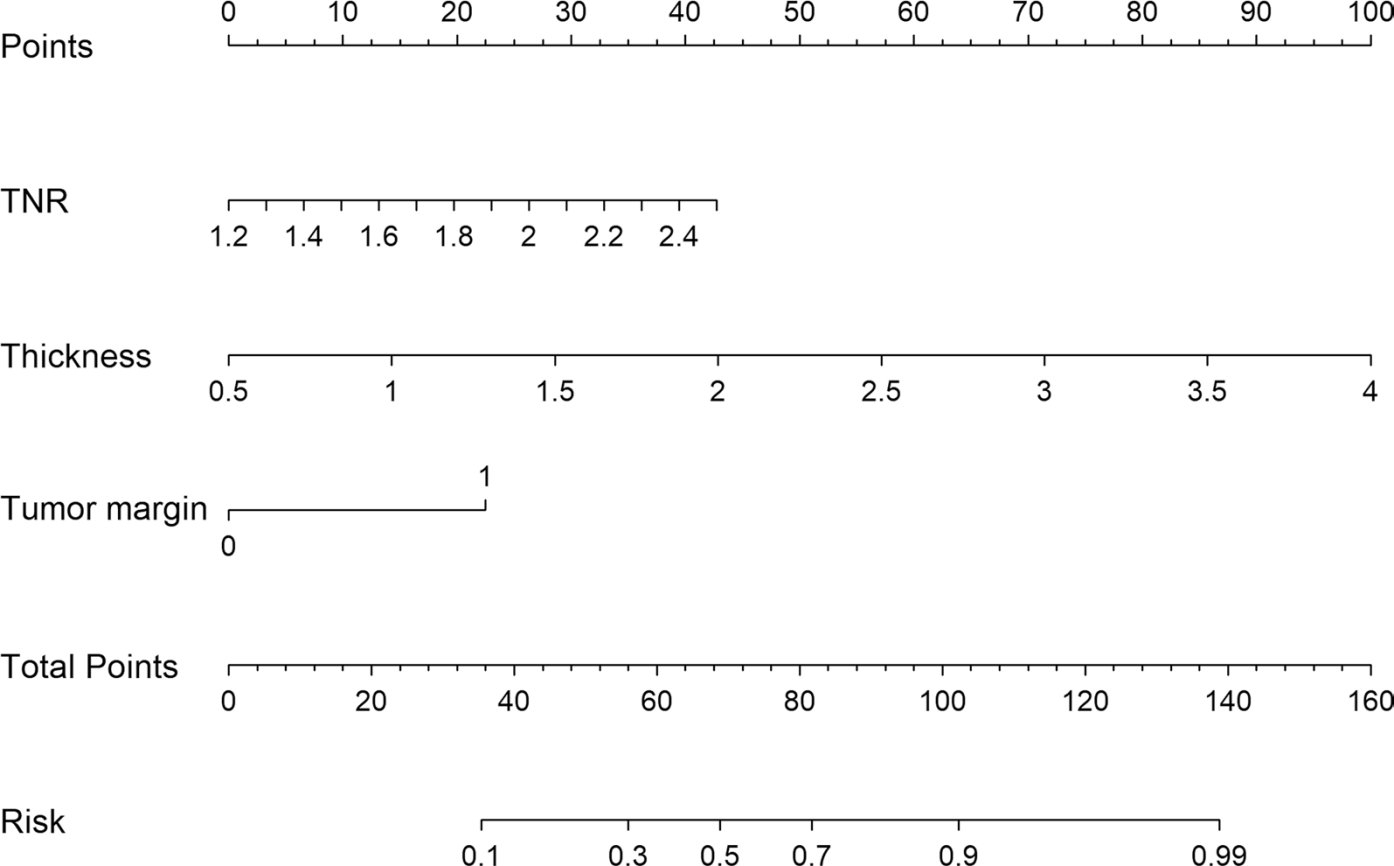
Nomogram for predicting LVI status in ESCC patients. The CECT-derived imaging feature predictive model is presented using a nomogram built with the TNR, Thickness, tumor margin, and score using a multivariable logistic regression model scaled by the proportional regression coefficient of each predictor. The ranges of TNR, Thickness, and tumor margin were 1.2 to 2.4, 0.5 to 4, and 0 to 1, respectively. The proportional regression coefficients of the TNR, Thickness, and tumor margin are scaled in the points. The points for the TNR, Thickness, and tumor margin are summed to obtain the total points in the points scale. The probability of LVI in ESCC patients is the corresponding number on the lower probability of risk scale

The diagnostic performance results of the TNR, Thickness, tumor margin and their combination are listed in Table 4. The ROC analysis results of the TNR, Thickness, tumor margin, and their combination for predicting LVI status in ESCC patients are shown in Fig. 5. Among the individual CECT-derived imaging features, Thickness had the highest AUC value (0.739, 95% CI 0.662–0.816), TNR had both the highest sensitivity (0.831, 95% CI 0.633–0.932) and NPV (0.892, 95% CI 0.843–0.907), and tumor margin had the highest accuracy (0.766, 95% CI 0.701–0.824), specificity (0.827, 95% CI 0.642–0.907) and PPV (0.618, 95% CI 0.516–0.662). The combination of the three features improved the AUC value (0.820, 95% CI 0.754–0.885), accuracy (0.797, 95% CI 0.734–0.851), specificity (0.848, 95% CI 0.652–0.928) and PPV (0.656, 95% CI 0.596–0.687). A nomogram was developed based on the multivariable logistic regression analysis (Fig. 6).

**Table 4:** Receiver operating characteristic analysis and diagnostic performance of TNR, Thickness, tumor margin, and their combination

Variables	AUC	Accuracy	Sensitivity	Specificity	PPV	NPV	Cutoff value
TNR	0.669 (0.594–0.745)	0.594 (0.522–0.663)	0.831 (0.633–0.932)	0.449 (0.312–0.565)	0.415 (0.341–0.433)	0.892 (0.843–0.907)	1.595
Thickness	0.739 (0.662–0.816)	0.716 (0.647–0.778)	0.644 (0.475–0.785)	0.708 (0.457–0.826)	0.519 (0.431–0.556)	0.842 (0.768–0.857)	1.545
Tumor margin	0.712 (0.642–0.782)	0.766 (0.701–0.824)	0.549 (0.380–0.698)	0.827 (0.642–0.907)	0.618 (0.516–0.662)	0.824 (0.780–0.833)	0.500
Combination	0.820 (0.754–0.885)	0.797 (0.734–0.851)	0.678 (0.525–0.780)	0.848 (0.652–0.928)	0.656 (0.596–0.687)	0.860 (0.826–0.871)	0.375

AUC, area under the curve; PPV, positive predictive value; NPV, negative predictive value

## Discussion

This study shows that preoperative arterial phase CECT-derived imaging features, which are obtained noninvasively, can predict LVI status in ESCC patients. However, accurate preoperative prediction of LVI status in ESCC can help clinicians choose better treatment strategies. The LVI status could effectively stratify the survival of lymph node-negative patients with ESCC, thus adding predictive value to the current TNM staging system [6]. Patients with negative lymph nodes may be upstaged in pathologic staging if the tumor presents with LVI. In clinical management, additional esophagectomy and lymphadenectomy are often recommended for ESCC patients with stage T1 after endoscopic resection if the postoperative pathology shows the presence of LVI [23]. Additional surgery may cause much more physical harm to the patient and increase the probability of complications. If we can accurately predict the LVI status in these patients, we can choose the proper treatment to avoid additional risks and complications. Meanwhile, patients with no lymph node metastases detected by CT but pre-operatively predicted to have LVI, aggressive lymph node dissection, extended surgical resection, or preoperative neoadjuvant therapy may be used to improve the prognosis [11, 12, 16, 24].

In this study, we found that tumors with LVI had higher CTV_Tumor_, TNR, and ΔTN values, greater Thickness, Length, and GTV, a higher proportion of heterogeneous enhancement and irregular margin, and were more likely to have EVFDT and tumor necrosis. The presence of these CECT-derived imaging features may indicate an increase in tumor aggressiveness and invasiveness, which corresponds to LVI status. The univariable analysis showed that CTV_Tumor_, TNR, ΔTN, enhancement pattern, Thickness, Length, GTV, tumor margin, EVFDT, and tumor necrosis were associated with the LVI status of ESCC patients. The multivariable analysis showed that the TNR, Thickness and tumor margin were independent predictors of LVI status in ESCC patients and had an acceptable predictive performance, and predictive performance could be improved by the combination of these predictors.

Contrast-enhanced CT images can improve the contrast between normal tissues and enhanced tumor margin, making it easy to distinguish the range of the primary tumor [25, 26]. Compared to nonenhanced images, CECT images may provide more detailed information about the tumor. In a previous study, Ma et al. [16] retrospectively analyzed preoperative CT images from 278 patients with AGC, including nonenhanced CT and multiple contrast-enhanced CT images (arterial phase, portal venous phase, and delayed phase). The results showed that the tumor CT attenuation difference between non-contrast and portal (ΔPP) and tumor-spleen attenuation difference in the portal phase (ΔT-S) were independent predictors of LVI status in patients with AGC. In another study, Yin et al. [17] retrospectively analyzed the preoperative precontract and dual-phase enhanced CT images of 64 patients with AGC. They found that the arterial phase contrast enhancement ratio (ACER) and the arterial parenchymal phase contrast enhancement ratio (APCER) were strongly associated with intratumoral microvascular and lymphatic invasion. Unlike the two studies mentioned above, the present study only included single arterial phase enhancement imaging features. Theoretically, the arterial phase, which reflects vascularity and hemodynamic changes, might reflect the presence of LVI [17]. Komori et al. [27] showed that the tumor-to-normal wall enhancement ratio in the arterial phase correlated with microvessel density and lymphatic vessel invasion. Similar to their study, we did not obtain noncontrast CT scans to assess the relative tumor enhancement ratio.

Our study showed that CTV_Tumor_, TNR and ΔTN were associated with LVI in the univariable analysis, but only the TNR was an independent predictor of LVI status in the multivariable analysis. The CTV_Tumor_ in the group with LVI was higher than the CTV_Tumor_ in the group without LVI, suggesting that the disruption of lymphatic vascular structures may increase microvascular permeability. On the other hand, this may suggest that LVI occurs when tumor cells infiltrate and destroy the vascular and/or lymphatic structures to form vascular cancer thrombi. Furthermore, tumor angiogenesis is characterized by an increase in the number of tumor blood vessels, and this process will impact CECT [28]. Due to the individual differences in the degree of enhancement during the arterial phase, the TNR may be a more stable predictor of LVI status.

A previous study confirmed that the enhancement pattern of the tumor could predict the histopathological type of the tumor, differentiate benign from malignant tumors, and reflect the pathological structure of the tumor [29]. Kim et al. [30] found that enhancement pattern analysis of arterially enhancing intrahepatic mass-forming cholangiocarcinomas can help differentiate them from hepatocellular carcinomas. In our study, the enhancement pattern of the tumors with LVI was more likely to be heterogeneous. This may be due to the high heterogeneity of tumors with LVI [16] and the histological component that determines the CT enhancement characteristics of the tumors [30, 31]. Thus, we can conclude that ESCC tumors with heterogeneous enhancement are more likely to develop LVI.

For conventional CT, the identification of esophageal carcinoma usually depends on finding esophagus wall thickening [32]. Umeoka et al. [25] found that arterial phase CECT images have the advantage of detecting lesions without wall thickening and show the best results in evaluating advanced lesions. In previous studies, larger diameter tumors have been shown to be a preoperative predictor of LVI in hepatocellular carcinoma patients [33].

For esophageal carcinoma, the thickness of the tumor measured on axial CT images represents the degree of tumor infiltration and is related to the T stage of the tumor [34, 35]. Another previous study showed that the mean postcontrast attenuation of the esophageal wall had a higher diagnostic performance in predicting pathological complete regression (pCR) than the maximum esophageal wall thickness [36]. However, in our study, Thickness had a higher AUC value and a lower OR value when predicting LVI status. This suggests that Thickness has a high predictive value before treatment of a tumor and that tumors with greater Thickness are more likely to have LVI.

Our study was the first to compare the relationship between Length, GTV, and LVI status in ESCC patients. As shown in Table 1, the tumors with LVI also had a greater Length and GTV than those without LVI. Kang et al. [18] found that the CT imaging-based tumor volume was superior to T staging for the depth of tumor invasion in predicting the prognosis of nonsurgical ESCC patients receiving definitive (chemo) radiotherapy. More importantly, GTV can predict postoperative survival, margin status, and lymph node positivity in esophageal carcinoma [37]. However, in the multivariable analysis, Length and GTV were not independent factors in predicting the LVI status in ESCC patients.

The present study revealed that the proportion of irregular tumor margin in the group with LVI was significantly higher than that in the group without LVI. In a previous study, Chou et al. [33] found that nonsmooth tumor margin is a promising two-dimensional imaging feature for preoperative assessment of microvascular invasion (MVI). In patients with renal cell carcinoma (RCC), the presence of an unsmooth margin or a finger-like protrusion is a CT-based tumor feature that can be used to predict pathologic renal sinus invasion (RSI) preoperatively [38]. These two studies suggest that tumor margin morphology has the potential to predict LVI status. Similarly, tumor margin had the highest accuracy, specificity, and positive predictive value in our study, and it can also be used as an independent predictor of LVI status in ESCC patients.

In addition, the CECT-derived imaging features that we studied included EVFDT and tumor necrosis. As a CECT-derived feature, EVFDT was present in 30.2% of gastrointestinal stromal tumors (GISTs), and patients with a primary lesion with EVFDT were likely to have higher risk stratification of GISTs than those without EVFDM (OR = 4.349, *P* < 0.05) [39]. The present study showed that EVFDT was present in 71.0% of the tumors with LVI, which was significantly higher than the proportion of tumors without LVI that had EVFDT (*P* < 0.05). This may be due to the presence of LVI, which increases the pressure on the small vessels supplying or draining the tumor, leading to dilatation of the lumen. For tumor necrosis, contrast-enhanced CT is considered the best modality for identifying necrosis [40]. A previous study found that more tumor necrosis and increased enhancement in adenosquamous carcinoma of the pancreas (PASC) may reflect the presence of a squamous component characterized by rapid proliferation and hypervascularity [41]. Similarly, we found that tumors with LVI had a higher incidence of necrosis, but necrosis was not an independent predictor, as revealed in the multivariable analysis.

There are several limitations to our study. First, this was a retrospective study, and there was some bias in the data collection due to missing data in some cases. Second, we did not perform precontrast and multiphase contrast-enhanced scans, which might result in a lack of some potentially valuable parameters. Third, as the esophagus is a hollow organ, the boundary between tumor tissue and normal esophagus is relatively difficult to determine; therefore, subjective errors may be introduced during the analysis. Finally, this study was a single-center study, and no multicenter validation was carried out, which we will focus on in a subsequent study.

## Conclusions

In conclusion, CECT-derived imaging features such as TNR, Thickness, tumor margin, and their combination can be used as preoperative predictors of LVI status for patients with ESCC. The results of this study may help to guide preoperative risk stratification to optimal treatment planning.

## Supplementary Information

**Additional file 1: Table S1.** The ICC analysis of intraobsever and interobsever agreements for quantitative features. **Table S2.** The Kappa analysis of intraobsever and interobsever agreements for qualitative features.

## Data Availability

The datasets used and analyzed during the current study are not publicly available due the inclusion of the patients' private data, but are available from the corresponding authors on reasonable request.

## Abbreviations

AUC: Area under the curve
CECT: Contrast-enhanced CT
CTV_Tumor_: CT attenuation value of the tumor
CTV_Normal_: CT attenuation value of the normal esophageal wall
ESCC: Esophageal squamous cell carcinoma
EVFDT: Enlarged blood supply or drainage vessels to the tumor
GTV: Gross tumor volume measured by CECT
HU: Hounsfield unit
ICC: Intraclass correlation coefficient
Length: Maximum length of the tumor measured by CECT
LVI: Lymphovascular invasion
ROC: Receiver operating characteristic
ROI: Region of interest
Thickness: Maximum thickness of the tumor measured by CECT
TNR: CT attenuation value ratio of the tumor-to-normal esophageal wall
