# Supplementary material for: Can lymphovascular invasion be predicted by contrast-enhanced CT imaging features in patients with esophageal squamous cell carcinoma? A preliminary retrospective study
Source: BMC Med Imaging. 2022 May 17;22:93. doi: 10.1186/s12880-022-00804-7 (PMC9116049; doi:10.1186/s12880-022-00804-7)
Supplement: Supplementary file 1 — Additional file 1: Table S1. The ICC analysis of intraobsever and interobsever agreements for quantitative features. Table S2. The Kappa analysis of intraobsever and interobsever agreements for qualitative features. [file 12880_2022_804_MOESM1_ESM.docx]

**Supplementary Information**

**Table S1.** The ICC analysis of intraobsever and interobsever agreements for quantitative features

| **ICC** | **CTV_Tumor_** | **CTV_Normal_** | **TNR** | **ΔTN** | **Thickness** | **Length** | **GTV** |
| --- | --- | --- | --- | --- | --- | --- | --- |
| Intraobsever | 0.936 | 0.930 | 0.985 | 0.971 | 0.969 | 0.955 | 0.985 |
| *P^1^* | 0.968^#^ | 0.337^#^ | 0.684^#^ | 0.776^#^ | 0.679^#^ | 0.989^#^ | 0.984^#^ |
| Interobsever | 0.919 | 0.915 | 0.986 | 0.968 | 0.937 | 0.912 | 0.925 |
| *P^2^* | 0.947^#^ | 0.945^#^ | 0.152^#^ | 0.664^#^ | 0.490^#^ | 0.757^#^ | 0.100^#^ |

ICC, intraclass correlation coefficient; ^#^Wilcoxon test; *P^1^*, Intraobsever; *P^2^*, Interobsever.

**Table S2.** The Kappa analysis of intraobsever and interobsever agreements for qualitative features

| **Kappa** | **Enhancement pattern** | **Tumor margins** | **EVFDT** | **Necrosis** |
| --- | --- | --- | --- | --- |
| Intraobsever | 0.908 | 0.935 | 0.848 | 0.862 |
| *P^1^* | 0.063^*^ | 0.063^*^ | 0.057^*^ | 0.737^*^ |
| Interobsever | 0.839 | 0.792 | 0.862 | 0.812 |
| *P^2^* | 0.727^*^ | 0.210^*^ | 0.267^*^ | 0.549^*^ |

^*^McNemar test; *P^1^*, Intraobsever; *P^2^*, Interobsever.
